# The impact of the transition from flipped classroom to online lectures on learning outcomes and student satisfaction in a rehabilitation medicine clerkship during the COVID-19 pandemic

**DOI:** 10.1186/s12909-022-03959-7

**Published:** 2022-12-20

**Authors:** Phichamon Khanittanuphong, Khanin Iamthanaporn, Jongdee Bvonpanttarananon

**Affiliations:** 1 Department of Rehabilitation Medicine, Faculty of Medicine, Prince of Songkla University, Hat Yai, Songkhla, Thailand; 2 Department of Orthopedics, Faculty of Medicine, Prince of Songkla University, Hat Yai, Songkhla, Thailand

**Keywords:** Flipped classroom, Online lectures, Clerkship, Rehabilitation, COVID-19

## Abstract

**Background:**

The flipped classroom (FC) is a well-known active learning module that activates the prior knowledge of students and promotes their cognitive skills during in-class activities. However, most on-site teaching during the COVID-19 pandemic had to be conducted online. The FC in our rehabilitation medicine clerkship curriculum was also shifted to online asynchronous lectures (OLs), without real-time interactions. There is no previous comparison of effectiveness between these two methods. Therefore, this study aimed to compare learning outcomes and student satisfaction in both FC and OL models.

**Methods:**

The study design was a historically controlled study. A physical modality was chosen for the content. The FC group (*n* = 233), in the academic years 2018 and 2019, was assigned to perform a pre-class activity consisting of reading study materials. Thereafter, the in-class activity comprised a small-group case-based discussion. The OL group (*n* = 240) in the academic years 2020 and 2021 followed an online model during the COVID-19 lockdown. They were also asked to read the online materials and then watch a self-paced recorded lecture video on Learning Management Systems. The learning outcomes, including their multiple-choice questions (MCQs) scores, final exam scores, grade points, and letter grades, were evaluated. Their overall course satisfaction ratings were also collected.

**Results:**

The OL group had an overall higher MCQ score for the physical modality portion than the FC group (*p* = 0.047). The median (lower quartile, upper quartile) of the total 50-MCQ scores were 34 (31, 37) in the OL group and 33 (29, 36) in the FC group (*p* = 0.007). The median final exam scores of the OL and FC groups were 69.5 and 68.3, respectively (*p* = 0.026). The median grade points and the letter grades were not significantly different between the groups. The proportions of satisfaction were significantly higher in the FC group than in the OL group.

**Conclusions:**

The OL group revealed significantly higher learning outcomes than the FC group. However, the FC group showed more satisfaction with interactivity than the OL group. The authors are of the view that a combination of both FC and OL methods will likely result in better outcomes.

## Background

In view of the rapid development of online technology and the use of the andragogical approach, which is a learner-centric concept used for adult education, a flipped classroom (FC) is a widely used educational method nowadays. This concept was proposed in medical education in 2012 [1]. It consists of two key steps. First, it is pre-class activity. Students are assigned to complete self-study of educational materials through a digital platform. Secondly, in-class activities are focused on clinical applications using case-based, problem-based, and team-based exercises. This process activates the prior knowledge of students [1]. The FC model aims to change traditional lectures or passive learning to active learning that promotes cognitive skills: analysis, synthesis, and evaluation by learners [2, 3].

Many original studies comparing the FC method with the traditional lecture method in health profession education have been published in recent years. Therefore, this study focused on previous meta-analyses and systematic reviews. A meta-analysis of 28 studies in 2018 revealed that the FC method has significant benefits compared with traditional classrooms for health professional education [4]. Ge et al. published a meta-analysis in 2020 that evaluated the effectiveness of FC and traditional lectures in radiology education [5]. They found that all outcomes, such as theoretic performance, practical skills, course satisfaction, teamwork ability, and self-directed learning, were significantly in favour of FC. Another review reported that students held positive perceptions of the FC approach [6]. However, there were inconclusive knowledge and skill changes because the median of the effect sizes was 0.08 with a 95% confidence interval of -0.21 to 0.21. A systematic review by Ramnanan et al. stated that medical students, at both preclinical and clinical levels, appreciated pre-class activities, which were easily accessible through online tools [7]. Overall, FC tends to have a positive effect on medical education.

In our curriculum, the FC model was applied to a rehabilitation medicine clerkship for the academic year 2018 to 2019. However, our faculty launched a new learning strategy during the COVID-19 pandemic in the academic year 2020 to 2021. Traditional lectures, including those under the FC model, were changed to online courses using the university’s Learning Management System (LMS). An in-class activity was modified from a face-to-face discussion to a self-paced online lecture combined with case scenarios to promote clinical application. Therefore, there was no interactivity between teachers and learners.

Tang et al. defined online lectures (OLs) as didactic lectures approached by any digital platforms that do not need active interaction with the video playback interface [8]. They reported that 45 studies showed high learner satisfaction and improvement in knowledge examination after availing the use of online lectures. This can possibly be attributed to the fact that students could independently access online materials anytime, learn at their own pace, and go back and repeatedly play such lectures. Camargo et al. published a review of online learning in medical schools during pandemic situations in 2020 [9]. They found that most studies applied pre-recorded methods rather than live sessions. However, these studies revealed no results regarding objective outcomes, such as final exam scores or numeric grade scores. Learning improvement was still unclear.

Previous evidence disclosed that both the FC and OL models presented some advantages for learning in medical education. To the best of the authors’ knowledge, however, there is no previous study comparing the effectiveness of these methods. Therefore, this study aimed to compare learning outcomes and student satisfaction in both the FC and OL methods in the context of a medical clerkship in rehabilitation medicine during the COVID-19 era.

## Methods

### Study design

The study design was a historically controlled study. The data were retrospectively collected from the database of the undergraduate medical education unit in the Department of Orthopedics, Faculty of Medicine, Prince of Songkla University, Thailand. The study was approved by the Human Research Ethics Committee (HREC) of the Faculty of Medicine at Prince of Songkla University (REC number: 65–233-11–1). The requirement for informed consent was waived by the committee.

### Participants

**Fig. 1:**
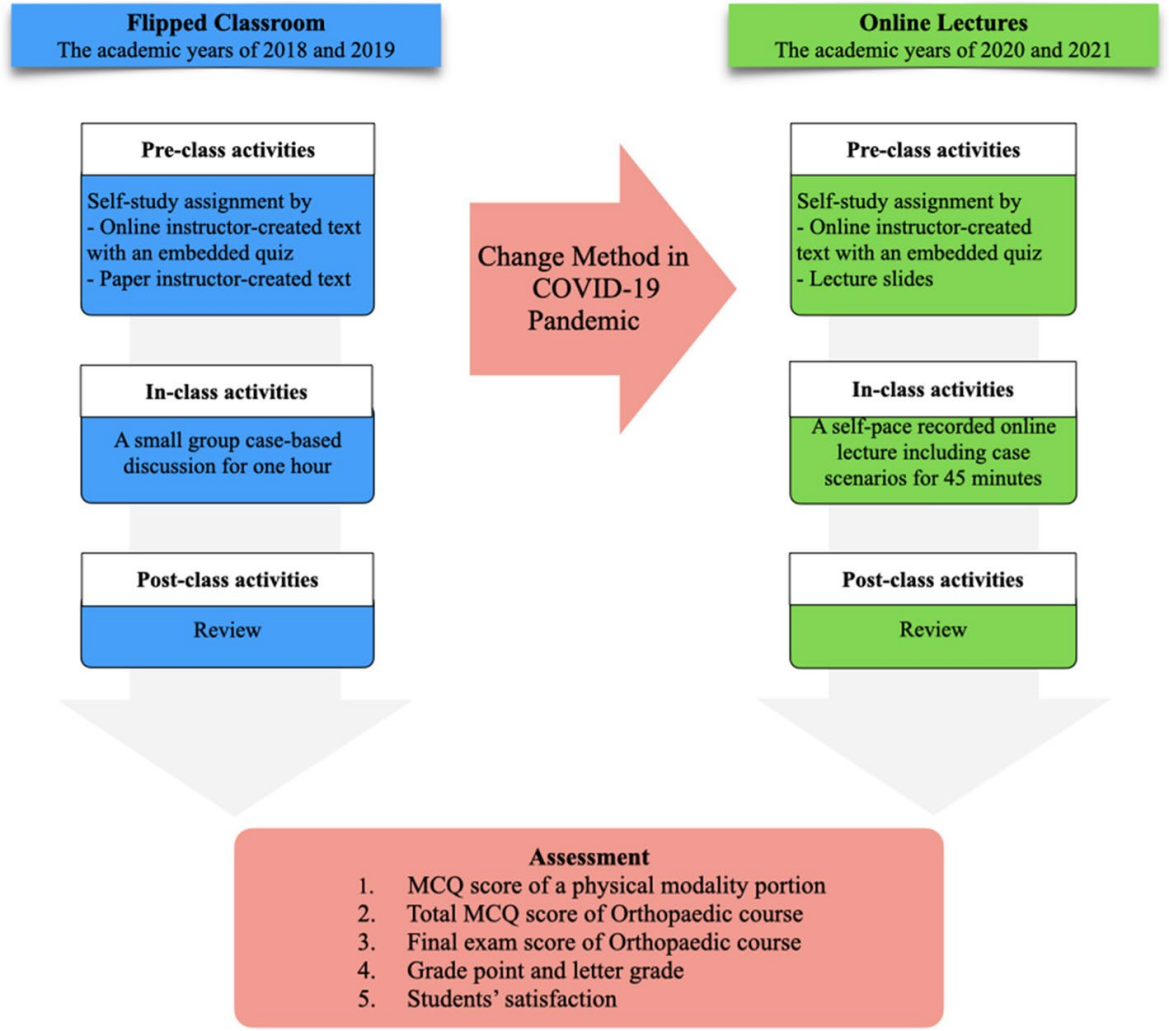
Flowchart of the flipped classroom and online lecture process

Participants recruited were 5th-year medical students who attended the rehabilitation medicine clerkship in the Orthopaedic course from 2018 to 2021. The FC model was used in the academic years 2018 and 2019. Thereafter, the model was changed to an online learning module in the academic years 2020 and 2021 due to the COVID-19 pandemic. The flowchart of both teaching processes is shown in Fig. 1.

### The face-to-face FC group

Students in the academic years of 2018 and 2019, prior to the COVID-19 pandemic, participated in the FC. All students were divided into twelve groups as per their academic year. Each group contained 10 to 12 students. The duration for each group on the Orthopaedic course was four weeks. The physical modality subject held in the first week of the course was chosen for this study. The FC consisted of three steps. First, it was a pre-class activity. The students were assigned to conduct self-study learning by reading an instructor-created text on paper or online material in the “Binla Book”, which was the official learning website of the faculty. These materials were provided four days before the in-class session. Second, the in-class activity was set for one hour. Case-based discussions were applied to draw on the prior knowledge of students, in a face-to-face interactive session among students. A teacher emphasised and summarised the clinical points. Finally, the post-class activity of the students was to review the overall learning content by themselves. The final examination was set in the fourth week of the course. It comprised 50 multiple-choice questions (MCQs) and covered all content in the Orthopaedic course, including 3 MCQs on the physical modality. Besides this, other assessments, including key features (KFs), modified essay questions (MEQs), and oral examinations, were also set. At the end of the course, the students were required to evaluate the teaching and their satisfaction with the FC.

### The online lecture group

The clinical curriculum was transformed to be taught on online platforms in the academic years of 2020 and 2021 because of the COVID-19 pandemic. Social distancing was required. Therefore, all traditional lectures, including the FC method, were changed to online asynchronous lectures accessible through the LMS. There still were three processes of learning, as in the FC method. First, the students were asked to prepare themselves through pre-class activities. Most study materials were the same as in the FC model. Lecture slides were also included. However, the second process, in-class activities, was different. Students were scheduled to watch a self-paced recorded lecture video on the LMS. The duration was 45 min. The lecture content contained some critical points and case scenarios. These were intended to make learners understand the relevant clinical practice. The students could contact a clinical instructor by email if they had any questions. Although this model is characterised by low interactivity, they could control the speed of the lecture, rewind, and fast-forward. Finally, a content review in post-class activities was designed to fulfil and complete their knowledge. The assessments were the same as in the FC model.

### Outcome measures

The primary objective was learning outcomes: (1) MCQ scores of a physical modality part; (2) total MCQ exam scores; (3) final exam scores; (4) grade point (out of 4); and (5) letter grades in the Orthopaedic course. The secondary outcome was student satisfaction. The assessment methods were classified into four levels according to the Kirkpatrick 4-level model [10]. The outcomes of this study are covered in levels 1, 2, and 3, as shown in Table 1.

**Table 1:** Outcome measures classified according to the Kirkpatrick 4-level model

Level assessment	Outcome measures
1 Reaction	Student satisfaction
2 Learning	3-MCQ scores of a physical modality part
3 Transferring of learning	Total 50-MCQ exam scores Final exam scores: sum of 50-MCQ, KF, MEQ, and oral exam Grade point Letter grades
4 Benefit to patients/Practice	None

*MCQ* Multiple-choice question, *KF* Key features, *MEQ* Modified essay questions

The Level 1 assessment was a reaction. It was defined as learner satisfaction or confidence. We collected responses regarding teaching evaluation and satisfaction after completing the course. This form was created by an undergraduate unit of the Department of Orthopedics. The identity of students was blinded to the clinical teacher to prevent informative bias towards students. There were four domains, including the learning environment, learning process management, teaching evaluation, and teacher, with a total of eight questions. Each question was evaluated by a numeric rating scale. The scale ranged from 0, which meant the least satisfied, to 4, which meant the most satisfied.

The Level 2 assessment was learning. It was defined as the knowledge of information directly taught in a lecture. This outcome was also recorded in this study. The MCQ with five options in the physical modality topic was examined. They consisted of three MCQs from a total of fifty MCQs. Question numbers were based on the duration of the lecture. The examination occurred two weeks after completing a physical modality hour. The MCQs were prepared in a paper-based examination for the FC. It was then changed to a computer-based examination, which was set on the LMS in the OL. All students in each group had to do the test at the same time using a computer at the Computer Assisted Instruction (CAI) hall of the faculty. The invigilator was on-site at the hall. Open books were prohibited.

**Fig. 2:**
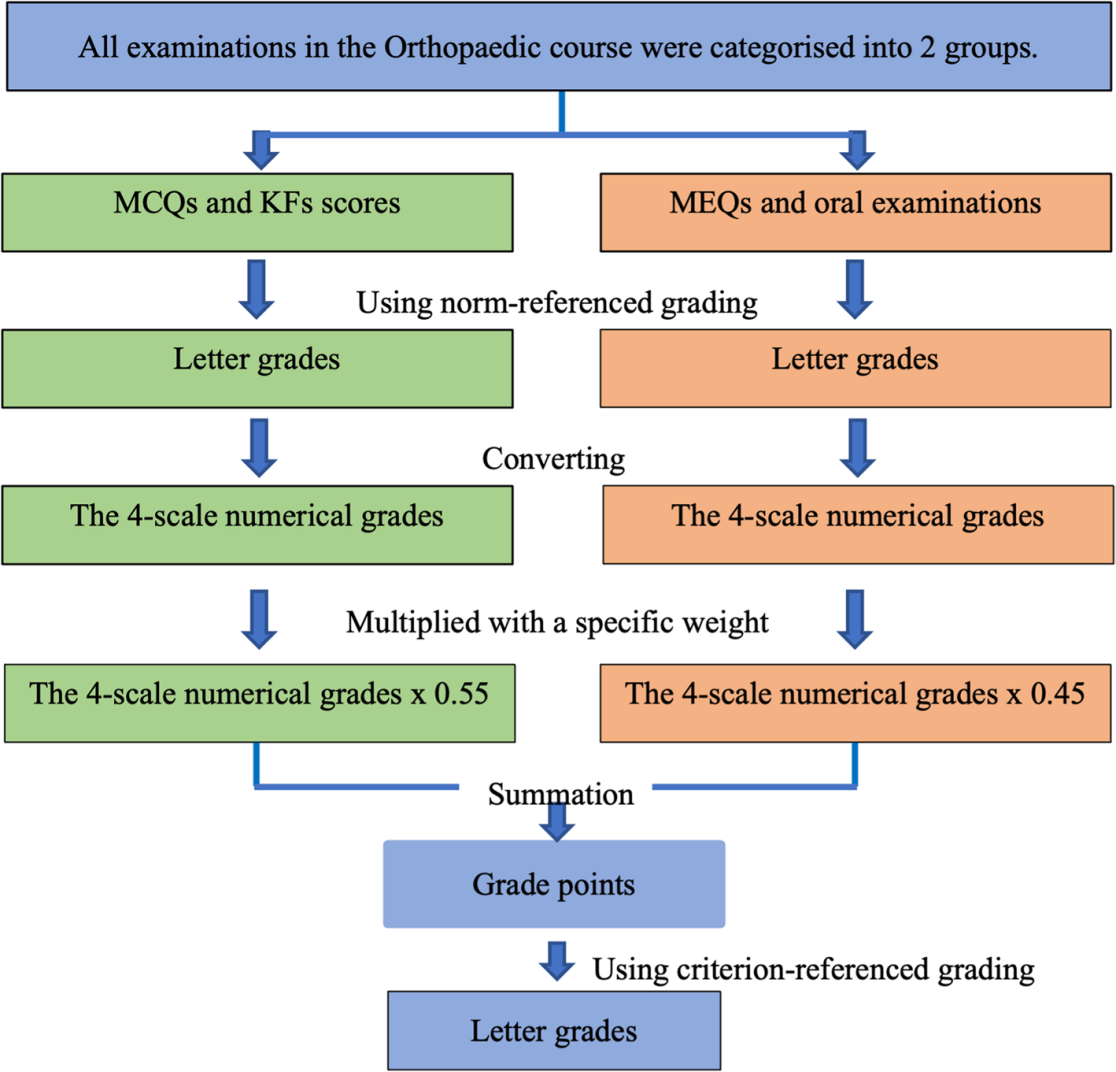
Flowchart of how to calculate grade points and letter grades of the Orthopaedic course

The Level 3 assessment involved the transferring of learning. Its definition included enhanced outcomes in tasks not directly taught in lectures, such as practical examinations or final course grades. The total MCQ exam scores, final exam scores, grade points, and letter grades were collected in our study. The total number of MCQs was fifty, including all contents of the Orthopaedic course. The final exam scores were summation from all tests: MCQs; KFs; MEQs; and oral examinations. The grade point was calculated by the specific method of our department (see Fig. 2). All assessments were divided into two parts: (1) MCQs and KFs; and (2) MEQs and oral examinations. The total scores of each part were converted to letter grades by norm-referenced grading. Each letter grade was assigned along with a numerical grade, for example, A to 4.00, B + to 3.50. Then, the numerical grade was multiplied with a specific score weight using 0.55 for the MCQs and KFs domain and 0.45 for the MEQs and oral examination domain. Next, the multiple of each part was summed to determine a grade point for the Orthopaedic course. Finally, the grade point was changed to a letter grade using criterion-referenced grading. Therefore, grade points and letter grades were not directly associated with final exam scores.

The Level 4 assessment, which was defined as a benefit to patients or organisational practice, was not evaluated in this study.

### Statistical analysis

The Shapiro–Wilk test was used to assess the normality of distribution for the tested variables. The distribution of all continuous data, such as 3-MCQ scores for the physical modality part, the total of 50-MCQ exam scores, final exam scores, and grade points, departed significantly from normality. Based on this result, the Wilcoxon rank sum test, which is a non-parametric test, was used. The median with lower and upper quartiles was presented for these variables. Categorical data, including letter grades and satisfaction, were presented as frequency (percentage). These variables were also analysed by the non-parametric tools. The chi-squared test was used for some domains of satisfaction, namely, instruction media, punctuality, and motivating learners. Fisher’s exact test was applied for the letter grades and the rest of the satisfaction domains. A *p* < 0.05 was considered statistically significant. The R software version 4.1.1 was used for statistical analysis.

## Results

**Fig. 3:**
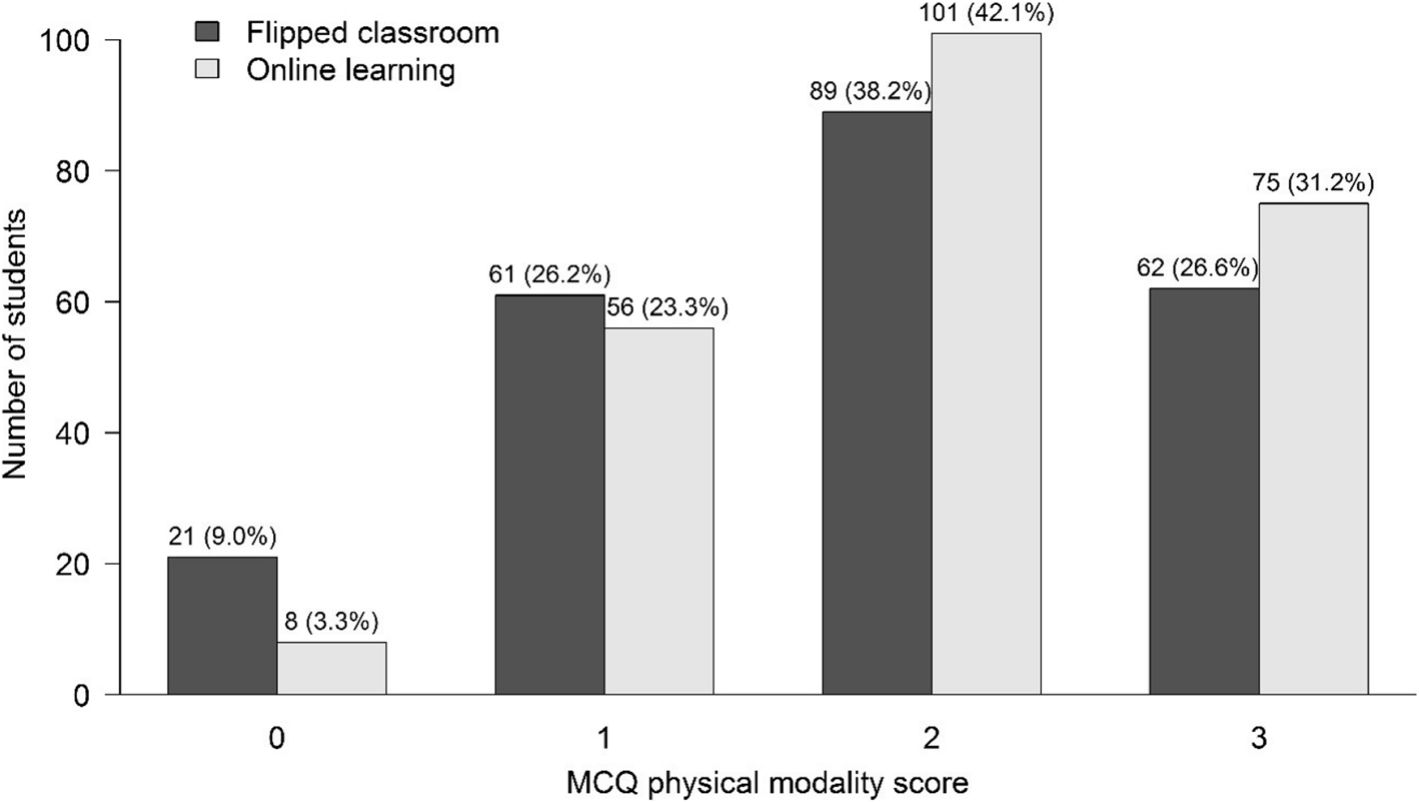
The number (percentage) of students was classified as a multiple-choice question (MCQ) score for the physical modality portion

**Fig. 4:**
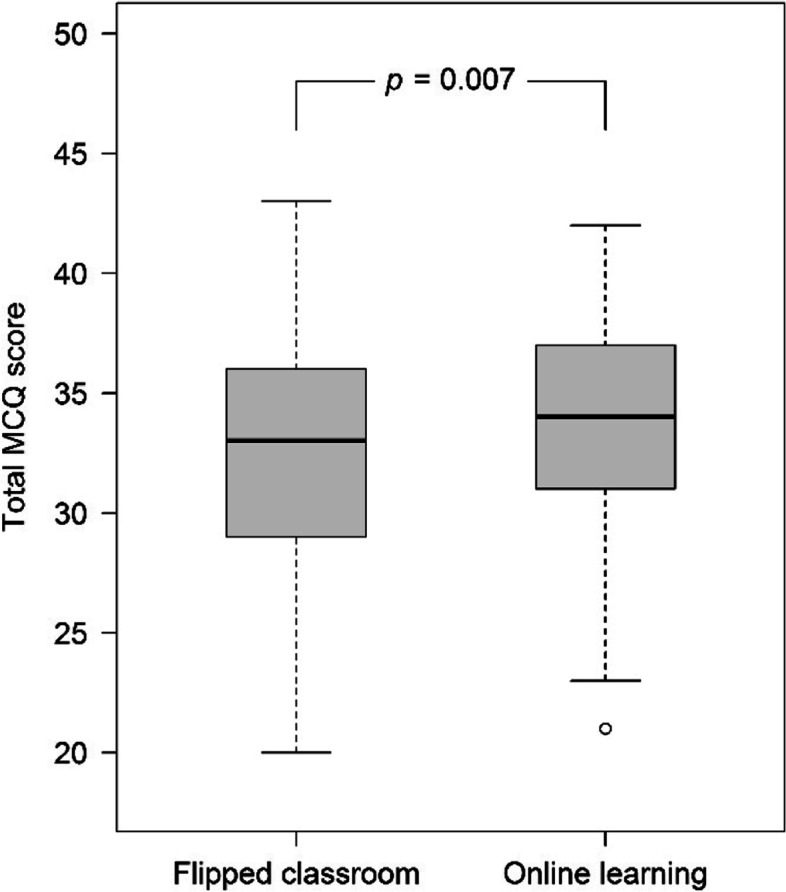
Comparison of median (lower quartile, upper quartile) of the total multiple-choice question (MCQ) scores between the groups

**Fig. 5:**
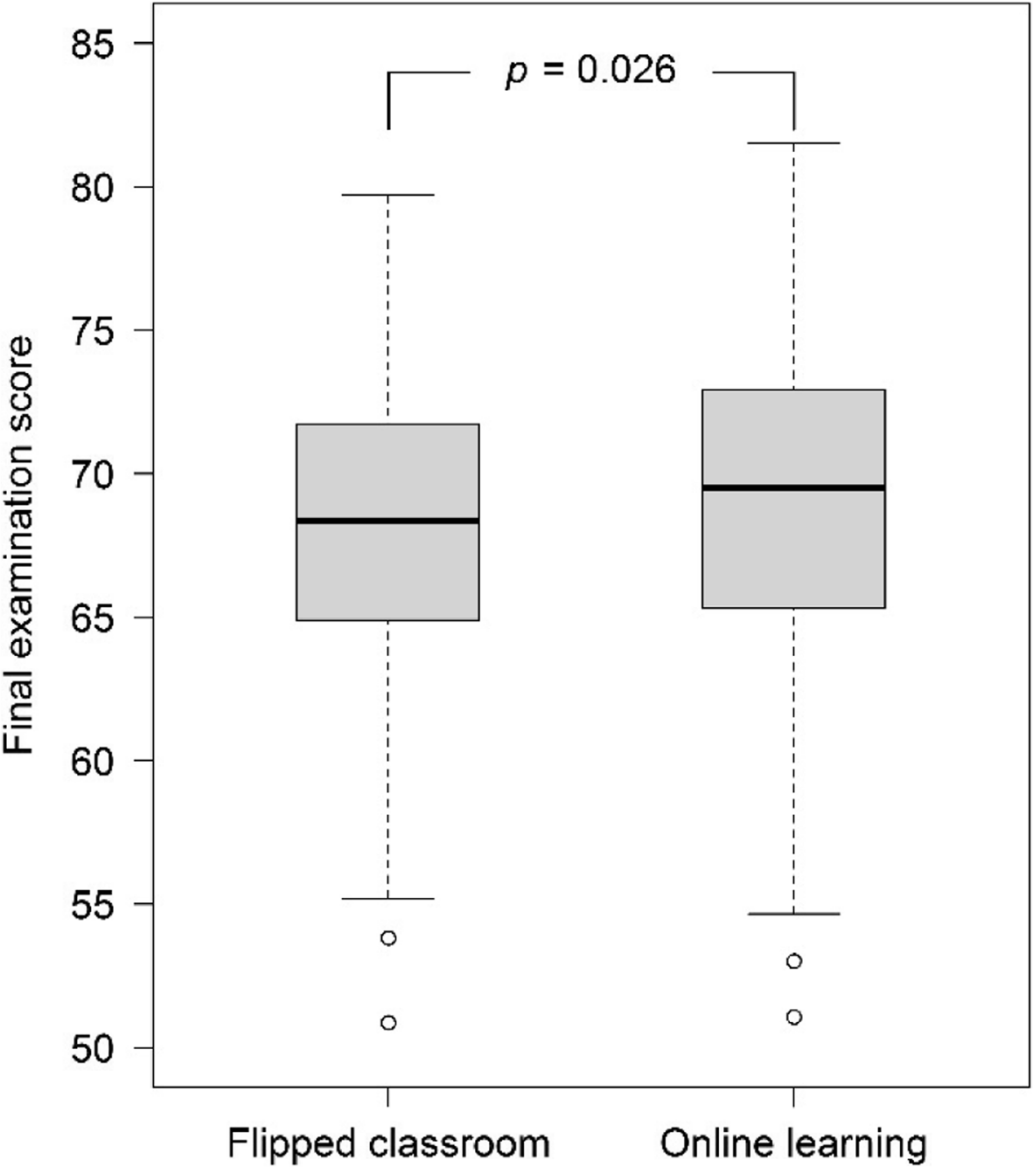
Comparison of median (lower quartile, upper quartile) of the final exam scores between the groups

The total number of learners in the FC and OL groups was 233 and 240, respectively. In terms of learning outcomes, the median MCQ score of the physical modality section was two from a total score of three in both groups (see Table 2). However, the FC group exhibited a higher percentage of scores of 0 and 1 than the OL group (see Fig. 3). Instead, the OL group showed that there was a higher percentage of scores 2 and 3 than the FC group (*p* = 0.047). The median total MCQ score was significantly superior in the OL group as shown in Table 2 and Fig. 4. The median final exam score was also significantly high in the OL group (Fig. 5). However, the median grade point and the percentage of the letter grades were not significantly different between the groups in Table 2.

**Table 2:** The learning outcomes

Variables	FC (n = 233)	OL (n = 240)	*p*-value
*Categorical data, n (%)*			
Letter grades			0.525
A	19 (8.2)	21 (8.8)	
B +	71 (30.5)	79 (32.9)	
B	64 (27.5)	53 (22.1)	
C +	59 (25.3)	68 (28.3)	
C	12 (5.2)	16 (6.7)	
X	7 (3.0)	3 (1.2)	
E	1 (0.4)	0 (0)	
*Continuous data, median (lower quartile, upper quartile)*			
MCQ score of physical modality portion (3 points)	2 (1, 3)	2 (1, 3)	0.037
Total MCQ score (50 points)	33 (29, 36)	34 (31, 37)	0.007
Final exam scores (0–100)	68.3 (64.9, 71.7)	69.5 (65.4, 72.9)	0.026
Grade point (0–4)	3 (2.7, 3.3)	3 (2.7, 3.3)	0.867

*FC* Flipped classroom group, *OL* Online lecture group

Finally, Table 3 shows the course evaluation and student satisfaction. The number of students (response rates) who replied to the course survey in the FC and OL groups was 225 (96.6%) and 232 (96.7%), respectively. The proportions of the levels of satisfaction in the FC group on the interactivity between teachers and learners significantly differed from the OL group. The other domains were insignificant.

**Table 3:** Course evaluation and students’ satisfaction

Domains	Most satisfied		Satisfied		Neither satisfied nor dissatisfied	
	FC	OL	FC	OL	FC	OL
1. Interactivity between a teacher and learners	209 (92.9)*	210 (90.5)	11 (4.9)	22 (9.5)	5 (2.2)	0
2. Learning process setting						
2.1 Accentuating learners’ participation	207 (92.0)	210 (90.5)	17 (7.6)	22 (9.5)	1 (0.4)	0
2.2 Application of instruction media	201 (89.3)	208 (89.7)	24 (10.7)	24 (10.3)	1 (0.4)	0
2.3 Organising the learning process that is applicable in a clinical situation	208 (92.4)	210 (90.5)	16 (7.1)	22 (9.5)	1 (0.4)	0
3. Assessment during teaching	202 (89.8)	204 (87.9)	22 (9.8)	27 (11.6)	1 (0.4)	1 (0.4)
4. Teacher						
4.1 Teaching method and personality	210 (93.3)	213 (91.8)	14 (6.2)	19 (8.2)	1 (0.4)	0
4.2 Punctuality	209 (92.9)	217 (93.5)	16 (7.1)	15 (6.5)	0	0
4.3 Motivating learners to express proper behaviour and valuing learners	208 (92.4)	215 (92.7)	17 (7.6)	17 (7.3)	0	0

Data are presented as n (%). *Significant different proportions (*p* = 0.01) between the two groups using Fisher’s exact test.

*FC* flipped classroom group, *OL* online lecture group

## Discussion

According to the examination, the OL group exhibited significantly higher MCQ scores and final exam scores than the FC group. However, grade points and letter grades had insignificant differences between the groups. This finding was possible because grade points and letter grades were not directly generated from the final exam score as described in the outcomes measure section. A review of undergraduate medical education reported that students who learned through online lectures had equal or better knowledge than students who learned by traditional methods, such as live lectures [8]. However, there was no comparison between the OL and FC models. Therefore, it is important for all aspects of both models, such as characteristics of each teaching method, learners, and study materials, to be considered.

First, there are several teaching models applied in medical education. The OL model was the most appropriate method during the COVID-19 pandemic, resulting from social distancing. The FC could be set up through an online program as well. However, the policy of the university’s medical education department determined that all lectures should be recorded and then be available online through LMS, under which interactivity could not be offered. The online model involved self-paced learning. It also delivered additional learning periods when learners could review lessons to gain a more in-depth understanding of the subject matter being taught. These measures improved learning outcomes [11]. In addition, time was constrained in clinical settings. The medical clerkship had both learning and clinical responsibilities. Therefore, the OL model that was easy to access at any time might have been a good method for such learners [12].

Physical modality, which was a subject in this study, was mainly a type of principal knowledge for medical students. The learning content was about the physiological effects of each therapeutic agent. The indication and contraindication of each modality were related to clinical application. However, learners did not need to practice by themselves. The OL model was probably appropriate for theory learning in that performance was not a predominant part. Therefore, the FC model might be a good option for skill training that needs a demonstration, performance of practical skills, and feedback [13, 14]. It was also found to be more suitable for problem-solving subjects than fact-based subjects requiring memorisation [15].

Second, learners might associate with learning outcomes. In light of the rapid changes in learning methods from traditional to online models, students were required to adapt their learning approaches and plan proper schedules [16]. This meant that self-regulated learners were activated by the COVID-19 pandemic [17, 18]. Many studies reported that students had positive perceptions of online lectures as a teaching technique [8]. Pre-class activities, such as reading or watching study materials, depended on students’ willingness in the OL. However, this is not an essential requirement like the FC model. Previous research reported barriers to reading assignments, such as time constraints, excessive volume of study materials, and examination preparation [19, 20]. Therefore, learners in the OL group experienced fewer difficulties than those in the FC.

Another concern was that learners did not know how to learn and how to adapt to the FC model in an effective manner [15]. This method has been successful when learners took responsibility for pre-class work and actively participated in in-class activities [15]. Insufficient pre-class preparation possibly resulted in learning outcomes [7]. Sabale and Chowdary revealed that learners who attended pre-class and in-class sessions obtained better examination scores than those who did not participate in any such portions [14]. Therefore, an instructor should introduce how to learn this model and the importance of pre-class assignments [20]. These points were also addressed as the nine principles of effective online flipped learning during the COVID-19 pandemic by Lo and Hew [21]. Principle 1 was the management of the proper transition to the new teaching method, including orientation to students. Adequate preparation time was supported by Principle 2 as well.

Third, study materials played a critical role for learners, especially in how they matched learning styles. The VARK model, which was proposed by Neil Fleming, was one of the widely used learning style approaches [22, 23]. It considered how persons’ senses assimilate information [24]. The abbreviated VARK stands for Visual, Aural/auditory, Read/write, and Kinaesthetic sensory modalities [21]. Previous research revealed that most medical undergraduates applied the multimodal type, which used a sensory approach for more than one mode, to enhance their learning [25–27]. Mainly multimodal types were bimodal learners in which auditory and reading were combined. In case they were the unimodal type, the predominant sense used was auditory [25]. The OL model in our study using these materials covered such learning styles more than the FC model. The authors prepared web-based video lectures for visual and auditory types and online instructor-created text for reading types in the OL. Instead, the FC only offered the text material. This issue was a weak point in the model. It was consistent with Principle 4 of Lo and Hew, which suggested using instructional videos to aid students’ pre-class learning in online flipped classrooms [21]. Some students expressed that learning through reading was uninteresting [28]. The advantages of a web-based video were the ability to control its playback based on the learner level, the reduction in learning burden by mentioning core content, and the ability to access it at any place and time [9, 28, 29]. Therefore, understanding learning styles aided instructors to created multimodal study materials [24]. In addition, the recommendations for pre-class learning materials were proposed, such as contributing the materials in advance, providing guidance for self-study, avoiding repeating materials from pre-class learning during in-class learning, concerning students’ preparation time, and beginning classes with students’ questions and summary of key points [30].

Aside from the learning outcomes, most students in the FC group, on the other hand, were more satisfied with the interactivity between teachers and learners than in the OL group. Preceding studies have reported similar results [7, 15, 29, 31]. The in-class activities of the FC model encouraged students to apply their knowledge from pre-class self-study to discuss case-based or clinical problems. Teachers had a role in feedback and direction [7]. Therefore, interactivity occurred among learners and teachers. Some research also revealed that healthcare students had positive perceptions of and satisfaction with the FC model [4–6]. In addition, students preferred small group classroom activities [7, 32]. This was also supported by Principle 8 of Lo and Hew [21]. Interaction among students would be well promoted by smaller group size, and the number of students per group in this study was ten to twelve. Students could thoroughly discuss the subject, and a teacher could react and comment accordingly, thereby covering all issues.

Some limitations of the study need to be reviewed. First, the outcomes lacked a performance examination such as an Objective Structured Clinical Examination (OSCE) or observation of clinical practice. Because the FC encouraged cognitive skills and clinical applications [6, 7], the MCQ examination could not completely assess these competencies. An assessment will encourage students to adapt their learning styles from being strategic learners, whose goals involve succeeding in only tests or MCQs, to being deep learners [25]. Next, the specific subtopics on the course evaluation and satisfaction, such as time-consuming pre-class activities, adequacy, a variety of study materials, and any barriers to an online system, were required to be evaluated. Open-ended questions about the advantages and disadvantages of the teaching methods, especially in an interactivity issue, were also an option. This information assisted with prevailing learning problems and with choosing suitable teaching methods. Finally, there were a small number of MCQ tests on the subject. The MCQs in the physical modality portion consisted of only three questions. The number of quizzes for each lesson was established by the undergraduate committee, depending on the teaching time. Pre- and post-test examinations were required to fill this gap.

## Conclusion

The OL model manifested significantly better learning outcomes compared with the FC model. However, the learners in the FC group showed more satisfaction with interactivity than those in the OL group. It is the authors’ view that a combination of both methods will likely make better outcomes. The OL could be merged into pre- and post-class activities for students’ preparation and review in the FC. In case of any pandemic occurs, the FC can perform using a proper online program along with various learning materials such as web-based recorded lectures and instructor-created texts. The distinctive points of both models can provide advantages for learners and help them be good doctors.

## Data Availability

The data and materials used and analysed during the study are available from the corresponding author upon reasonable request.

## Abbreviations

FC: Flipped Classroom
OL: Online Lecture
LMS: Learning Management System
